# Zur Bedeutung von *Places* und Landschaften für die Gesundheit

**DOI:** 10.1007/s00103-025-04116-3

**Published:** 2025-08-26

**Authors:** Thomas Kistemann, Timo Falkenberg

**Affiliations:** 1 Institut für Hygiene & Public Health, Universitätsklinikum Bonn, Venusberg-Campus 1, 53127 Bonn, Deutschland; 2 Geographisches Institut der Universität Bonn, Bonn, Deutschland

**Keywords:** Therapeutische Landschaft, Salutogenese, Gesundheitsgeografie, Cultural Turn, Spatial Turn, Therapeutic landscape, Salutogenesis, Health geography, Cultural turn, Spatial turn

## Abstract

Das Konzept der Therapeutischen Landschaften hat sich in den letzten Jahrzehnten als analytischer Rahmen zum besseren Verständnis der Bedeutung von spezifischen Orten und Landschaften für Gesundheit und gesundheitliches Wohlbefinden empirisch bewährt. Es basiert auf der Grundannahme, dass ein jeweils *Place-*spezifisches Set ökologischer, sozialer und kultureller Merkmale räumlicher Settings messbar Gesundheitswirksamkeit entfaltet, und bietet als Theorie die Möglichkeit, der empirischen Komplexität der phänomenalen Welt eine konzeptionelle Ordnung zu geben. Zum ideengeschichtlichen Hintergrund zählen Salutogenese, Gesundheitsförderung, *Cultural Turn* und *Spatial Turn*. Ansätze zur Erklärung der Wirkentfaltung Therapeutischer Landschaften basieren auf der Akteur-Netzwerk-Theorie (ANT) sowie dem auf der *Theory of Mind* basierten Konzept des Mentalisierens. Während zunächst außergewöhnliche Orte und Heilungseffekte im Fokus standen, geht es inzwischen eher um Alltagsorte und diverse gesundheitsrelevante Wirkungen.

## Abstract (en)

The concept of therapeutic landscapes has proven itself empirically in recent decades as an analytical framework for better understanding the significance of specific places and landscapes for health and health-related well-being. It is based on the fundamental assumption that a specific set of ecological, social and cultural characteristics of spatial settings has a measurable effect on health. As a theory, it offers the possibility of providing a conceptual order for the empirical complexity of the phenomenal world. The background in terms of the history of ideas includes salutogenesis, health promotion, the cultural turn and the spatial turn. Approaches to explaining the development of therapeutic landscapes are based on the actor-network theory (ANT) and the concept of mentalising, based on the “theory of mind”. While the focus was initially on extraordinary places and healing effects, it is now more about everyday places and diverse health-relevant effects.

## Einleitung

Für die Analyse physischer, sozialer und symbolischer Faktoren, die an ausgewählten *Places, *d. h. mit Bedeutung aufgeladenen Orten (im Gegensatz zu *Spaces*), wirken und die sowohl physische als auch psychische Gesundheitswirksamkeit entfalten, bietet das Konzept der Therapeutischen Landschaften einen analytischen Rahmen. Als „Therapeutische Landschaft“ wird dabei die Summe der Qualitäten und Valenzen von geografischen Orten bezeichnet, die in einem sehr umfassenden Sinne gesundheitswirksam sind [1]. Ihre Wirkung leitet sich nicht nur aus ihren physischen Qualitäten ab, sondern umfasst auch die immateriellen Eigenschaften und Zuschreibungen. Doch wo liegen die Gründe für die erfolgreiche Rezeptionsgeschichte des Konzepts, welches Gesler [2] vor über 3 Jahrzehnten einführte? Mehrere zusammenwirkende Aspekte sind hierfür erkennbar, die einerseits eng mit der Kritik des die Medizin dominierenden biomedizinischen Krankheitsmodells und andererseits mit der Rezeption der Neuen Kulturgeografie durch die Medizinische Geografie zusammenhängen [3].

Unsere Arbeit stellt das Konzept der Therapeutischen Landschaften vor. Zunächst werden Aspekte des ideengeschichtlichen Hintergrunds behandelt. Im Anschluss werden das Konzept, empirische Erfahrungen, konzeptionelle Erweiterungen sowie theoretische Ansätze zur Erklärung der Wirkentfaltung vorgestellt. Vor einem knappen Fazit wird dargelegt, dass begründet von einer Theorie der Therapeutischen Landschaften gesprochen werden kann.

## Ideengeschichtlicher Hintergrund

### Relativierung des biomedizinischen Krankheitsmodells

Seit der zweiten Hälfte des 19. Jahrhunderts dominiert ein naturwissenschaftliches Paradigma die Medizin. Krankheit wird dabei als Abweichung von einem natürlichen Zustand des Organismus gesehen, hat eine bestimmte Ätiologie und Prognose. Im Ergebnis erlaubt dieses biomedizinische Krankheitsmodell die dichotome Unterscheidung von „krank“ und „gesund“. Seine Schwächen liegen in der Annahme einfacher Ursache-Wirkungs-Beziehungen sowie im Ausblenden sozialer, kultureller und psychischer Einflüsse.

Neue Krankheits- und Gesundheitsmodelle haben im Laufe der letzten Jahrzehnte wesentliche Kritikpunkte an der biomedizinischen Modellfamilie aufgegriffen und Alternativen entwickelt. Der wissenschaftliche Diskurs wird dabei im Wesentlichen von nichtmedizinischen, randmedizinischen und gesundheitswissenschaftlichen Disziplinen getragen. Eine bedeutsame Neuorientierung stellte das Modell der Salutogenese dar [4], da es Gesundheit und nicht Krankheit in den Mittelpunkt der Betrachtung stellt. Wesentlich ist einerseits die Annahme, dass Gesundheit und Krankheit Pole eines Kontinuums sind, und andererseits das Prinzip der Heterostase, der zufolge Krankheiten normale Erscheinungen des Lebens und nicht Abweichungen von der Normalität sind. Die Bewegung auf dem Kontinuum wird durch Widerstandsressourcen zum Umgang mit Stressoren bestimmt; diese können gesellschaftlicher (politisch, ökonomisch, sozial) und individueller Art (kognitiv, psychisch, physiologisch, materiell) oder umweltbezogen sein. Erfolgreicher Umgang mit Stressoren führt zu einem durchdringenden Gefühl des Vertrauens (Kohärenzgefühl), welches sich aus den Teilaspekten Verstehbarkeit und Handhabbarkeit interner und externer Stimuli sowie Sinnhaftigkeit des eigenen Lebens konstituiert. Im Zuge der Weiterentwicklung des Salutogenesemodells wurde ein Strang des ressourcenfördernden Erlebens und Verhaltens ergänzt: Inneres Gleichgewicht, ausgeglichene Stimmung, Humor, Optimismus sowie die Fähigkeit zu verzeihen fördern die Gesundheit [5].

Das Salutogenesekonzept überwindet wesentliche Beschränkungen des biomedizinischen Paradigmas, indem es die empirisch nicht belegte Dichotomie von Gesundheit und Krankheit ablehnt. Es schafft die Theoriebasis für eine Gesundheitsförderung, die auf Stärkung gesellschaftlicher und individueller Ressourcen zielt. Gesundheitsorientierte Modelle berücksichtigen auch die vielgestaltigen Interaktionen von Menschen mit ihrer physischen, sozialen und kulturellen Umwelt. Und diese Interaktionen finden notwendigerweise an Orten statt, womit die räumliche Dimension gesundheitsrelevant wird: „*Places matter!*“ [6].

### Gesundheitsförderung

Die Ottawa-Charta der Weltgesundheitsorganisation (WHO; [7]) bildete die Grundlage eines neuen Primats der Gesundheitsförderung. Dabei geht es um Maßnahmen, die Individuen befähigen, ihre Gesundheit durch ihr Verhalten zu erhalten und zu unterstützen. Als ein vorrangiges Handlungsfeld der Gesundheitsförderung wurde die Schaffung gesunder Lebenswelten benannt. Die WHO rief dazu auf, dass die Art und Weise, wie Gesellschaften Arbeit und Freizeit organisieren, Quelle von Gesundheit und nicht von Krankheit sein sollte. Gesundheitsförderung hat in diesem Sinne die wesentliche Aufgabe, sichere, anregende, befriedigende und angenehme Lebensräume zu schaffen. Die Ottawa-Charta ist auch ein Meilenstein für die Integration von Gesundheitsförderung in städtebauliche Leitbilder. Erstmals adressierte die WHO damit die Bedeutung räumlicher Bedingungen für die Gesundheit.

### *Cultural Turn*

In der Humangeografie setzte in den 1980er-Jahren ein Diskurs über die Rezeption des sogenannten *Cultural Tur*n ein. Mit diesem Begriff werden in den Kulturwissenschaften mehrere recht unterschiedliche Phänomene bezeichnet: Kulturelle Forschungsgegenstände rücken in den Mittelpunkt, kulturelle Einflüsse auf Wirtschaft und Gesellschaft werden berücksichtigt und vereinfachende strukturalistische Erklärungsansätze werden kritisch gesehen. Vielmehr geht es um die umfassende Analyse konkreter, einzigartiger Gegenstände unter Verwendung qualitativer Forschungsmethoden [8].

Kultur wird als Chiffre für gesellschaftlich institutionalisierte Sinnwelten aufgefasst, innerhalb derer die Menschen denken und handeln. Maßgeblich für die Entwicklung einer „Neuen Kulturgeografie“ war ein interpretativer Kulturbegriff, wonach Kultur ein vom Menschen gesponnenes Bedeutungsgewebe ist, das wie ein Text zu lesen, zu analysieren und interpretieren ist [9]. Der *Cultural Turn* impliziert eine kulturalistische Verschiebung in mindestens vier Dimensionen: Auf der ontologischen Ebene gibt es eine Verschiebung von der Annahme einer beobachterunabhängigen Realität zu einer sozial konstruierten und kulturell vorinterpretierten „Wirklichkeit“. Auf der epistemologischen Ebene tritt die (kritische) Rekonstruktion und Reinterpretation von Handlungen und Sinnordnungen an die Stelle eines positivistischen Wissenschaftsbegriffs. Methodisch ergänzen teilnehmende Beobachtung, qualitative Interviews und Diskursanalysen Kartierungen, Zählungen und standardisierten Befragungen. Die vierte Dimension bezieht sich auf die Inhalte, z. B. die Untersuchung der kulturellen Konstitution sozialer Milieus [8].

### *Spatial Turn*

Im Kontext der kultur- und sozialwissenschaftlichen Beschäftigung mit der Frage der sozialen Konstituierung von Raum wurde von Soja [10] der Begriff *Spatial Turn* eingeführt. Damit wurden auch Positionen der einflussreichen französischen Philosophen Foucault und Lefebvre wieder aufgegriffen: Jeder Raum ist im Sinne eines dynamischen, aktiven und politisch aufgeladenen Prozesses gesellschaftlich produzierter Raum. Er wird nicht exklusiv als Behälter verstanden, sondern als das Ergebnis sozialer Beziehungen und kultureller Aktivitäten, als sozial und kulturell wirksame Größe. Der euklidische Raum wird ergänzt durch sozial und kulturell überformte Raumwahrnehmungen und -konstruktionen. Das Räumliche wird damit zur eigenen Analysekategorie. Für die geografische Gesundheitsforschung bietet der *Spatial Turn* die Chance, sich als Raumwissenschaft innerhalb der Medical Humanities zu profilieren [11].

### Reformierte Medizinische Geografie

Medizinische Geografie untersucht als Wissenschaft an der Schnittstelle von Medizin und Geografie traditionell die räumlichen Dimensionen von Gesundheit und Krankheit mit geografischen Konzepten und Methoden. Vor dem Hintergrund der oben dargestellten Entwicklungen forderte Kearns [12], die Medizinische Geografie grundlegend zu reformieren. Er sah in der Verbindung von humanistischer Geografie und modernen Gesundheitsmodellen den Beginn einer „post-medizinischen“ Geografie der Gesundheit. Die Emanzipierung vom biomedizinischen Modell, die kulturwissenschaftliche Neuorientierung, die sozialtheoretische Fundierung und das politische Engagement für Public Health fanden ein breites Echo [13].

Unter dem Eindruck von *Humanistic Turn, Structuralistic Turn *und *Cultural Turn *wurde die Agenda der Medizinischen Geografie erweitert: Der *Humanistic Turn* bildete sich seit den späten 1980er-Jahren in Arbeiten zu Gesundheit und gesundheitsbezogenem Verhalten heraus, welche simplifizierende normative Verhaltensannahmen ablehnten. In den Mittelpunkt des Interesses rückten die Erforschung gelebter Gesundheitserfahrungen sowie die soziale Konstruktion von Gesundheit und Krankheit. Der *Structuralistic Turn* stellt die ökonomischen, sozialen und politischen Determinanten für die Verteilung von Gesundheitschancen und Gesundheitsdienstleistungen in den Mittelpunkt der Forschung. Insbesondere beschäftigt er sich mit den Interaktionen zwischen der individuellen Ebene sowie den strukturellen und materiellen Zwängen, unter denen die Gesundheitserfahrungen der Menschen geprägt werden. Der *Cultural Turn* schließlich ergänzte die kulturelle Perspektive und markierte damit die Entwicklung zu einer Geografie der Gesundheit. „*Culture matters*“ [14, S. 34]! Die Bedeutung von Raum und Ort *an sich* für Individuen und ihre Gesundheit trat in den Fokus.

## Therapeutische Landschaft

### Das Konzept

Gesler [2] erkannte, dass aus den Kategorien der Neuen Kulturgeografie ein großer Nutzen für das Verständnis gesundheitswirksamer Prozesse gezogen werden kann, und integrierte diese in den Begriff *Therapeutic Landscape. *Diese Bezeichnung verwendete er als eine geografische Metapher, die zu verstehen hilft, wie sich gesundheitswirksame Prozesse an gewissen *Places* (Settings, Situationen, Standorten, Milieus) entfalten. Das Konzept vereint Aspekte der Humanökologie, des Strukturalismus und des Humanismus, die als physische, soziale und symbolische Faktoren operationalisiert werden. Dieser facettenreiche kulturgeografische Landschaftsbegriff erlaubt, Landschaften verschieden zu sehen und zu interpretieren:

- naturalistisch, indem Landschaften als humanökologisch wirksame Mensch-Umwelt-Wechselbeziehung verstanden werden;
- humanistisch, indem Bedeutung, Erfahrung, Subjektivität und Symbolwert berücksichtigt werden;
- strukturalistisch, indem Landschaften als soziale, gesellschaftlich produzierte Konstrukte interpretiert werden;
- poststrukturalistisch, indem Landschaften als diskursive Konstruktionen gedeutet werden.

Diese Landschaftsinterpretationen überlagern sich als Schichten einer Therapeutischen Landschaft; die relative Bedeutung verschiedener Therapeutischer Landschaftsschichten variiert situativ [15] und auch zwischen unterschiedlichen Nutzergruppen: Die Ausdeutung Therapeutischer Landschaften ist letztlich abhängig von persönlichen Haltungen und Erfahrungen [16].

Therapeutische Landschaften, insbesondere Alltagsorte, reichen von solchen mit offensichtlich hohem ästhetischen Wert bis zu solchen, deren gesundheitswirksame Qualitäten für einen Außenstehenden mitunter gar nicht wahrnehmbar sein können. In diesem Zusammenhang erweist sich das „*Sense-of-Place*“-Konzept als zentral für das Verständnis individueller Erfahrungen von Wohlbefinden. *Sense of Place* bezeichnet die Bedeutung, die Intention, die Wertung und den Stellenwert, den Menschen *Places *geben [17].

Die oben genannten 4 *Wirklichkeits*dimensionen von Therapeutischen Landschaften ergänzten Völker und Kistemann [18] um 4 *Aneignungs*dimensionen zu einem 2‑dimensionalen Modell Therapeutischer Landschaften. Diese Aneignungsdimensionen bezeichnen die Weise, in welcher Menschen mit den Wirklichkeitsdimensionen von Landschaft agieren: in kontemplativem Erfahren und Erleben, in physischer Aktivität, in sozialer Interaktion oder als Gegenstand symbolischer Aufladung. Zudem haben Therapeutische Landschaften einen dynamischen Charakter. Sie spiegeln veränderliche Umwelten (Witterung, Tages‑, Jahreszeiten) ebenso wider wie Veränderungen der medizinischen Versorgung oder den Wandel der sozialen Konstruktion von Krankheit – „*History matters!*“ [19]. Insofern bildet Zeit die 4. Dimension Therapeutischer Landschaft.

Die Therapeutische Landschaft, als komplexes Modell komplementärer Bedeutungsebenen [15], als Summe der gesundheitswirksamen Dimensionen von *Places*, wurde zum Paradigma einer erneuerten Geografie der Gesundheit. Es steht für das Verständnis, dass Gesundheit und Gesundheitsversorgung an *Places* stattfindet, die beackert und gefühlt werden, die eine Haltung veranlassen und repräsentiert werden [20], die bedeutsam sind für Gesundheit.

### Erfahrungen und Erweiterungen

In den vergangenen 30 Jahren haben zahlreiche empirische Untersuchungen die Beobachtung unterstützt, dass Landschaften „therapeutisch“ sind, d. h., dass sie sich auf Gesundheit und Wohlbefinden auswirken. Gesler [2] hatte in seinen empirischen Untersuchungen zunächst *Places* im Visier, die explizit die *Heilung* von Krankheit unterstützen, und interessierte sich für *Places* außerhalb des täglichen Lebens [21]. Williams [22] erweiterte das Konzept, indem sie auch *Places* einbezog, die Gesundheit *fördern* und erhalten. Die gesundheitsfördernden Qualitäten von Therapeutischen Landschaften umfassen unter anderem stressabfedernde Mechanismen, Moderation von Stress und Angst [23], gesteigerte Zugehörigkeit und Bestimmung [24], positive Stimmungsregulation [23], körperliche Aktivität [25] sowie soziale Interaktion und Förderung des sozialen Kapitals [26]. Trotz seiner pathogenetischen Konnotation wurde der Begriff „Therapeutische Landschaft“ beibehalten.

Die räumliche Dimension Therapeutischer Landschaften kann ganz unterschiedlich und flexibel gewählt werden: eigener Körper, Zuhause, Park, Stadt etc. Und auch die Erfahrungen und das Verständnis jedes Einzelnen unterscheiden sich je nach geografischen, kulturellen, sozialen und individuellen Merkmalen [27].

Weil Menschen dort mehr Zeit verbringen, ist das gesundheitswirksame Potenzial von Alltagsorten (*Everyday Landscapes*) wahrscheinlich größer als das von außergewöhnlichen Orten [13, 28]. Deshalb wurden zunehmend auch Alltagsorte, wie etwa Bibliotheken [29] oder die eigene Wohnung [30], als Therapeutische Landschaft untersucht. Zuhause konstruieren Menschen ihre Alltagsorte als Therapeutische Landschaften und kombinieren dabei unterschiedliche Strategien: z. B. Reduzierung der Exposition gegenüber nachteiligen Agentien (z. B. Lärm), Schaffung persönlicher Räume, soziale Unterstützung durch Mitbewohner [30].

Doughty [31] plädierte für eine Mobilisierung des Konzepts der Therapeutischen Landschaften (*Mobility Turn*): Sie rezipierte die Metapher von der Therapeutischen Landschaft als dynamischen und relationalen Prozess, als beweglichen Raum, der sich eher in und durch Interaktion mit der Umwelt entfaltet, als dass er ein fester Platz sei: eher orts*bewusst* als orts*gebunden*. Gatrell [32] stellte die gesundheitswirksame Qualität des Bewegungsvorgangs an sich in den Mittelpunkt und entwickelte ein eigenes Konzept von *Therapeutic Mobilities*, das drei Elemente umfasst: körperliche Aktivität, soziale Aktivität und den räumlichen Kontext des Gehens (*Walkability*). Beim gemeinsamen Wandern wird Therapeutische Landschaft durch mobile Auseinandersetzung mit Orten produziert.

## Theorien zur Wirkentfaltung

Umweltpsychologische Theorien zur Erklärung des gesundheitswirksamen Potenzials von *Places* und Landschaften gehen davon aus, dass bestimmte Landschaftsformen einen evolutionären Überlebensvorteil boten, das „genetische Gedächtnis“ prägten [33, S. 25] und deshalb bevorzugt werden. Zu ihren Schwächen gehört, dass interindividuelle Unterschiede weitgehend unberücksichtigt bleiben.

Im Mittelpunkt der Forschung zu erklärenden Mechanismen der Wirkung Therapeutischer Landschaften stehen seit Längerem subjektorientierte Analysen von Gesundheitserfahrungen. Dabei werden materiellen Eigenschaften, psychophysiologischen, sozialen, ästhetischen und relationalen Dimensionen gesundheitswirksame Eigenschaften zugeschrieben [16]. Menschen erleben Landschaften in unterschiedlicher Weise und Erfahrungen mit *Places* resultieren stets aus spezifischen Formen der Auseinandersetzung. Insofern lässt sich die Erfahrung einer Therapeutischen Landschaft am ehesten als eine relationale Wirkung erfassen, die sich durch Interaktionen zwischen Individuum und Landschaft manifestiert. Diese Interaktionen umfassen sowohl die unmittelbare körperliche Erfahrung als auch deren spätere Interpretation. Das Bedürfnis, mit der Diversität von Farben, Formen und Texturen, die in Grenzräumen am größten ist, zu interagieren, ist hierbei offensichtlich besonders bedeutsam [34]. Zwei Erklärungsansätze fanden in den letzten Jahren besondere Beachtung.

Conradson [16] greift für seine theoretische Fundierung auf die Akteur-Netzwerk-Theorie (ANT) zurück, nach der die Welt netzwerkartig verfasst ist, deren Bestandteile sich als mehr oder weniger kohärente Akteure aus verschiedenen Elementen zusammensetzen. Aktion ist das Produkt spezifischer Netzwerkverbindungen, die einen Akteur räumlich und zeitlich mit einem anderen verbinden. *Places* fungieren als Knoten in relationalen Netzwerken [35]. Thrift [36] fokussierte mit seinem Konzept der „*Ecology of Place*“ auf die räumliche Dimension: *Place* ist demnach eine aktive und konstitutive Präsenz, die Einstellungen und Interaktionen formt und erdet. Und auch *Places* bilden sich durch Interaktionen heraus – zwischen Menschen, zwischen Menschen und Dingen, zwischen Menschen und anderen Lebewesen. Alle Beziehungen, welche das relational konzipierte menschliche Selbst formen, besitzen typischerweise auch geografische Einbettung und räumliche Konsistenz. Diese (therapeutische) Landschaftserfahrung umfasst sowohl physiologische als auch interpretative Elemente [16]. Netzwerkrelationen sind Ressourcen, welche die Realisierung bestimmter Aktionen und die Erlangung bestimmter Handlungsfähigkeiten (*Agencies*) unterstützen [37]. Und *Places* sind das Medium zur Generierung und Verteilung dieser Ressourcen. Da sich Gesundheit, salutogenetisch verstanden, aus Ressourcen konstituiert, müssen für das Verständnis von Gesundheitsförderung sowohl diejenigen Ressourcen erfasst werden, die für die Entfaltung gesundheitsbezogener Kräfte erforderlich sind, als auch die spezifischen *Places*, an denen diese Ressourcen verfügbar sind bzw. (re)generiert werden können. Die Konstitution Therapeutischer Landschaften kann man sich insofern vorstellen als Zusammenspiel von Relationen, Ressourcen und Handlungsfähigkeiten. Duff [37] unterscheidet soziale, affektive und materielle gesundheitswirksame Ressourcen. Ihre Eigenschaften sind relationale Errungenschaften: Sie resultieren aus jeweils einzigartiger Konvergenz und Relation gesundheitswirksamer Ressourcen an einem spezifischen *Place*. Es geht dabei nicht um die Identifizierung von „Idealtypen“; vielmehr muss dem Zusammenspiel vielfältiger Prozesse, durch die soziale, affektive und materielle Ressourcen an Alltagsorten generiert werden, Beachtung geschenkt werden [38].

Rose [39] hingegen geht in ihren theoretischen Überlegungen von der Praxis, Landschaft zu *sehen*, aus. Unsere Praktiken des Sehens reproduzieren Räume (*Spaces*) als spezifische *Places*. Es ist wichtig, was das Objekt in die Begegnung einbringt und was der Seher: Das Objekt „antwortet zurück“ [39]. Der Betrachter von Landschaft ist nicht nur kognitiv, sondern auch emotional und affektiv engagiert. Erst Letzteres bildet die Basis für Erfahrungen, die zu einem Gefühl des Wohl- oder Missbefindens beitragen. Aber wie kommen derartige Effekte zustande? Hier greift Rose [39] auf das Konzept des *Mentalising* im Rahmen der *Theory of Mind* zurück [40]: einen Mechanismus des emotionalen Spiegelns, erklärt durch das soziale Feedback-Modell reflektierter Affekte [41]. „Mentalisieren“ bezeichnet unsere Fähigkeit, Bewusstseinsvorgänge und Verhalten eines sozialen Gegenübers durch Zuschreibung der zugrunde liegenden emotionalen Zustände zu deuten.

Rose [39] verbindet das Konzept des „Mentalising“ mit der affektiven Erfahrung beim Betrachten einer Landschaft. Insofern wird das Konzept auf die individuelle Interaktion mit einem Objekt (Landschaft) angewendet. Rose postuliert, dass ein Individuum Landschaft als einen „empathischen Spiegel“ erfahren kann, der das Verständnis der emotionalen und kognitiven Bedingungen des subjektiven und intersubjektiven Funktionierens unterstützt: Indem wir einem *Place* (einer Landschaft) gleichzeitig als objektive Realität und als Repräsentation begegnen, können durch diesen *Place* Emotionen und Kognitionen empathisch zu uns zurückgespiegelt werden und dadurch einen Prozess des Mentalisierens auslösen. *Places* durch Visualisierung, Imagination und eben Mentalisieren einen Sinn zu geben, das ist eine essenzielle, überlebensnotwendige Fähigkeit: Um eine komplexe visuelle Situation zu verarbeiten, kreieren wir mentale Repräsentationen [34]. In gewisser Weise ist die Beziehung zwischen Menschen und *Places* oder Landschaften als Resonanzphänomen zu sehen, angesichts dessen „die Welt den handelnden Subjekten als ein antwortendes, atmendes, tragendes, in manchen Momenten sogar wohlwollendes, entgegenkommendes oder ‚gütiges‘ Resonanzsystem erscheint“ [42, S. 9].

Analog zum Feedback-Mechanismus zwischen Kleinkind und Eltern kann ein Individuum, indem es Landschaftsmanifestationen als „Gesicht“ aufnimmt, eigene emotionale und kognitive Zustände darauf projizieren, in der Wiederbegegnung wiedererkennen und reproduzieren: Diese Auseinandersetzung kann dann auch Grundlage gesundheitsrelevanter Erfahrung sein. Sehweisen, Visualisierung, Ab-Bildung, neben anderen sensorischen Erfahrungen wesentlich im Prozess des Mentalisierens, sind nicht allein auf individuelle Biologie reduzierbar, sondern vielmehr auch kulturell konstruiert. Im Prozess des Mentalisierens von *Places* entwickeln und verstärken wir tiefe Erfahrungen des „Verortetseins“ und frischen Bedürfnisse, Gefühle und Sehnsüchte auf [34]. Das Konzept des Mentalisierens bietet einen Erklärungsansatz dafür, wie gewisse gesundheitswirksame Effekte auftreten können.

In diesem Konzept findet das Paradigma der Therapeutischen Landschaft auch Anschluss an den *Posthumanistic Turn*, der für die Sozialwissenschaften in den letzten beiden Dekaden bedeutsam wurde. Damit wird eine Reihe humanistischer Grundannahmen infrage gestellt, unter anderem die der Autonomie der Menschen, deren Existenz und Handlungen nicht abhängig seien von anderen, nichtmenschlichen Entitäten [43]. Gleichzeitig korrespondiert es mit dem Entwurf eines dreidimensionalen Persönlichkeitsmodells, das die Bedeutung der menschlichen Umwelt (soziales Gegenüber) ebenso berücksichtigt wie die der nichtmenschlichen Umwelt [44].

Zur Interaktion zwischen *Places* und menschlichem Gehirn liegen inzwischen auch umfangreiche neurowissenschaftliche Erkenntnisse vor [45]. Das Gehirn verfügt über hochspezialisierte Strukturen (Hirnareale, Subregionen, Zellarten) und Prozesse zur Verarbeitung räumlicher Information. Dem Hippocampus fällt dabei eine zentrale Rolle zu [46]. Analogien fallen auf zwischen *Place Cells* und Spiegelneuronen, die das Verhalten des sozialen Gegenübers spiegeln: *Places* werden wiedererkannt und wirken auf das Wohlbefinden, weil bei ihrer Wahrnehmung und Erinnerung stets auch emotionale Anteile des limbischen Systems stimuliert werden.

## Vom Konzept zur Theorie

Das Konzept der Therapeutischen Landschaft hat sich in den letzten Jahrzehnten als ein „theoretischer Dreh- und Angelpunkt“ [47] bewährt, der methodische Experimente erleichtert, eine Rückkehr zur idiografischen Tradition der Medizinischen Geografie ermöglichte und eine Plattform bot, um weiterführende theoretische Überlegungen anzustellen. Das Konzept basiert auf der Grundannahme, dass das jeweils spezifische Set ökologischer, sozialer und humanistischer Merkmale räumlicher Settings sowohl quantitativ als auch qualitativ messbare Gesundheitswirksamkeit entfaltet. Insofern kann es im Sinne von Suddaby [48] auch als „Theorie“ angesprochen werden, welche eine Möglichkeit bietet, der empirischen Komplexität der phänomenalen Welt eine konzeptionelle Ordnung zu geben. Sie bietet ein System wissenschaftlich begründeter Aussagen, das geeignet ist, Gesetzmäßigkeiten zu erklären und Prognosen zu erstellen. Als „verstehende Theorie“ zielt sie darauf ab, Phänomene nicht auf ihre kausalen Beziehungen zu reduzieren, sondern sie im Kontext der von Menschen erlebten und zugewiesenen Bedeutungen im hermeneutischen Sinne zu *verstehen* [49]. Als Theorie ist das Konzept der Therapeutischen Landschaften konsistent, empirisch verankert, besitzt Erklärungswert und ermöglicht Prognosen. Der Korpus empirischer Befunde stützt die Formulierung von Verallgemeinerungen, die zu prognostizieren erlauben, was bei einem gegebenen Input beobachtet werden wird. Indem die Theorie etablierte Denkweisen hinterfragt und alternative Sichtweisen auf Phänomene anbietet, kann sie auch als „provokative Theorie“ angesprochen werden [49].

Zusammengefasst ergibt sich eine Theorie der Therapeutischen Landschaft, die sowohl mit der ANT (*Places* als aktive und konstitutive Netzwerkknoten) als auch der Theorie des Mentalisierens (*Places* als Emotionen und Kognitionen widerspiegelnde Repräsentationen) harmoniert. In ihrer Mitte steht die Trias aus *Place*, Identität und Gesundheit. Im Spannungsfeld der Konstitution von Identität in einer stetig sich wandelnden Lebenswelt kann Therapeutische Landschaft als Moderator interpretiert werden, der dieses Paradoxon aushaltbar macht.

Spätestens seit dem *Spatial Turn* und dem damit verbundenen konstruktivistischen Raumverständnis beeinflusst Foucault auch die Raumwissenschaften. Daran anknüpfend bietet sich an, als Untersuchungsraster Therapeutischer Landschaften sein „Dispositiv“ zu nutzen: das Netz, das zwischen einem heterogenen Ensemble von Elementen geknüpft werden kann, welche für eine bestimmte Zeit und eine bestimmte Gesellschaft bedeutsam sind. Zu diesen „Elementen“ des Dispositivs gehören Diskurse, Institutionen, architekturale Einrichtungen, reglementierende Entscheidungen, Gesetze, administrative Maßnahmen, wissenschaftliche Aussagen und Lehrsätze [50, S. 119 f.].

## Fazit

Das gesundheitsgeografische Paradigma der Therapeutischen Landschaften reflektiert die kulturalistischen Verschiebungen in den Sozialwissenschaften und fußt auf einer holistischen Konzeption von Gesundheit. Es erlaubt eine mehrdimensionale Interpretation der gesundheitsrelevanten Interaktionen von Menschen mit *Places* und Landschaften. Zunächst standen vor allem *heilende* Wirkungen außergewöhnlicher *Places* und Landschaften mit natürlichen bzw. inszenierten Naturschönheiten im Mittelpunkt des Interesses. Heute geht es insbesondere um Alltagsorte und deren *gesundheitswirksame *Effekte. Die Theorie der Therapeutischen Landschaft kann hilfreich sein, um Gesundheit auch im Kontext großer und aktueller Themen wie Bevölkerungswachstum, Globalisierung, Ökonomisierung, Urbanisierung, Biodiversitätsverlust, Postkolonialismus, Mobilität, Kommunikation und Umweltgerechtigkeit zu sehen, einzuordnen und zu interpretieren.

Neben der Akteur-Netzwerk-Theorie bietet die *Theory of Mind* mit dem Konzept des *Mentalising* einen konsistenten Ansatz zum besseren Verständnis der Zusammenhänge zwischen *Places *und Gesundheit: Indem wir einem *Place* sowohl als Realität als auch als Repräsentation begegnen, können durch diesen *Place* Emotionen und Kognitionen zurückgespiegelt werden. Das „Gesicht“ des *Place*, der Landschaft, ist das personale Gegenüber, mit dem wir interagieren. Als Untersuchungsraster Therapeutischer Landschaften bietet sich Foucaults „Dispositiv“ an.
